# Competitive integrated employment for youth with disabilities: Aligning measurement with conceptualization using latent growth curve modeling

**DOI:** 10.1371/journal.pone.0290573

**Published:** 2023-11-16

**Authors:** Sayed Mostafa, Yudan Chen Wang

**Affiliations:** 1 Department of Mathematics & Statistics, North Carolina Agricultural and Technical State University, Greensboro, NC, United States of America; 2 Department of Counseling, North Carolina Agricultural and Technical State University, Greensboro, NC, United States of America; Guangxi Normal University, CHINA

## Abstract

**Purpose:**

Although existing research has generated a wealth of information related to employment for individuals with disabilities, a major limitation is that common measurements of employment do not fully capture the scope of the optimal outcome specified in public policies, namely, competitive integrated employment. Therefore, we aimed to describe the change and stability in multiple aspects of employment for youth with disabilities from high school age to mid-30s under the structural equation modeling framework.

**Methods:**

We identified a sample of 1,921 youth with disabilities who were at least 18 years old in 2003 from the National Longitudinal Survey of Youth– 1997. We estimated a series of latent growth curve models to assess trajectories of job quality, indicated by hourly pay, and stability, indicated by weekly work hours, over a period of 15 years.

**Results:**

Trajectories of job quality and stability did not covary or load on a common factor, but there was substantial variability within the sample in both the intercept and slope of these two constructs, which were best captured by a cubic growth curve, and partially explained by health condition and several demographic variables.

**Conclusions:**

Competitive integrated employment comprises of multiple components which should ideally be considered along a time dimension. Future studies need to assess validity of the measurement model with a different sample and incorporate another important component of competitive integrated employment, that is, whether work is carried out at an integrated setting.

## Introduction

In the 2018–2019 school year, about 14% of all public-school students ages 3 to 21 received special education services (SES) under the Individuals with Disabilities Education Act [IDEA; 1]. For the >7 million youth with disabilities [2], long-term inclusion and integration into society with economic and social self-sufficiency are the hope of these individuals and their families, with competitive integrated employment being the marker of success as defined by social services and policies [3].

The series of federal regulations related to vocational rehabilitation (VR) for persons with disabilities, starting from the Rehabilitation Act of 1973 to the most recent Workforce Innovation and Opportunity Act (WIOA) in 2015, is intended to encourage persons with disabilities to engage in skills training and eventually competitive integrated employment via the protection and support of educational institutions, state VR agencies, and local career centers. Research on the long-term outcomes of youth with disabilities found that job training and work experiences during high school were positively associated with employment outcomes five years post-high school [4]. Additionally, postsecondary education support remained beneficial nine years after the VR application [5]. Moreover, services from these various sources are most effective when they are offered in a coordinated manner [6, 7].

The benefits of VR support before and during transition from school to work for youth with disabilities, however, do not seem to translate into improvement in labor force statistics, as the labor force participation rates and employment-population ratios for adults with disabilities have remained stagnant in the past decade [8]. Since WIOA was implemented only a few years ago, it may take more time for its impact to emerge at the population level. On the other hand, we believe the effects of VR services for transitional youth will be more accurately captured by employment trajectories based on repeated measurement of the same individuals than by cohort employment rates. There are two reasons for this argument. First, disability status is a fluid characteristic [9]. Health statistics show that the prevalence of disability increases with age. As such, the population who contribute to the annual labor participation statistics for the 25–44 age group is not a continuation of the 18–24 age group. Many individuals in the older age group have adult-onset disabilities and hence did not benefit from VR services during school-to-work transition. Second, employment status is also a fluid and multi-dimensional characteristic [10, 11]. As a key feature of competitive integrated employment is stability–not necessarily remaining in the same position but remaining in the labor market–an appropriate indicator for optimal employment should be a stable trajectory of being gainfully employed instead of a snapshot of employment status, even though the snapshot approach has frequently been used in many previous studies. Previous research on the long-term outcomes of youth with disabilities rarely goes beyond age 30. More importantly, researchers seldom consider the dynamic nature of employment status. Thus, it is critical to take into account the multi-dimensional nature of employment so as to examine the effectiveness and efficiency of intervention programs and social policies. The lack of such knowledge will impede the effort to coordinate VR services at different levels, both horizontally (across agencies serving the same age groups) and vertically (across agencies serving different age groups).

### Defining and measuring competitive integrated employment

Under WIOA, the optimal outcome of VR services is competitive integrated employment, meaning full or part-time work at minimum wage or higher, with wages and benefits comparable to the customary rate paid to employees without disabilities performing the same work, at a location where the employee with disabilities interacts with coworkers without disabilities, and with opportunities for advancement similar to other employees without disabilities in similar positions [12, 13]. In this sense, stability is also an essential feature of competitive integrated employment, as advancement would not happen without stability. Thus, this conceptualization of competitive integrated employment requires that a proper evaluation of employment outcomes should capture the multi-dimensional nature of each position holistically and longitudinally.

In practice, each state has to report rate of engagement in competitive integrated employment as one of the post school outcomes for students who enrolled in special education [e.g., 14], following the definition under IDEA of 2004 that “competitive integrated employment occurs when a student has worked for pay at or above the minimum wage in a setting with others who are nondisabled for a period of 20 hours a week for at least 90 days at any time in the year since leaving high school and includes military employment.” As such, there is potentially a great deal of variability in the extent of integration, in the amount of pay, and in the number of work hours among those who meet the mark of competitive integrated employment. Moreover, information on the stability and prestige of employment is unlikely to be property documented administratively.

The fluid and multi-dimensional nature of employment is rarely examined in research studies. Instead, employment tends to be treated as a binary variable–whether someone was employed at the time of data collection [e.g., 14] or whether someone was ever employed during a period of time [e.g., 4]. For example, studies utilizing the RSA-911 data generally used successful case closure, or closure with competitive integrated employment, as the outcome. Due to the fact that the RSA-911 system discontinues data collection once a case is closed, employment can only be recorded with a snapshot. Nonetheless, the schedule and quality of employment are likely to fluctuate over time, particularly for persons with disabilities. Longitudinal datasets such as the National Longitudinal Survey of Youth– 1997 (NLSY97) afford the opportunity to examine simultaneously all aspects of employment, including weekly work hours and wages, but this full set of elements were not leveraged in many research studies using this dataset.

### Predicting competitive integrated employment

Although the operationalization and measurement of employment outcomes vary across different studies, a general understanding is that, with proper and sufficient support, youth with disabilities are capable of living productive lives. Nonetheless, existing research has found that the employment outcomes of youth with disabilities are influenced by personal and family characteristics. Both being male and being White are associated with higher odds of having competitive employment since high school [15–17]. Education level, or amount of education received, is positively associated with VR case closure in young adulthood [15] and mid-adulthood [18]. Across the age spectrum, Brucker and Henly [19] found that being older than 24, having a high school education or above, being White, and being in excellent or very good health led to higher probability of obtaining a job above the median wage with benefits, regardless of disability status. Additionally, family socioeconomic conditions were significantly associated with education and employment prospects. According to Wagner et al. [17], when annual household income was below $50,000 or when the head of household had less than high school education, youth with disabilities were less likely to attend postsecondary education or vocational programs. Further, youth from low-income families were less likely to have any competitive employment after high school. The link between family socioeconomic conditions and vocational outcomes remained present even after controlling for a series of individual characteristics such as type of impairment and level of functioning.

### The present study

Although existing research has generated a wealth of information on how employment outcomes are predicted by individual, family, school, and community factors, a major limitation is that the measurement of employment does not fully capture the scope of optimal outcomes specified in policies such as WIOA or IDEA. Competitive integrated employment features equitable pay, stability, and an integrated setting. As such, we ought to consider multiple indicators of employment longitudinally to determine whether “competitive integrated employment” is achieved.

To bridge the gap in existing literature with regard to the measurement of competitive integrated employment, we utilized the NLSY97 data to describe the change and stability in two elements of employment, namely, pay and work hours, from high school age to mid-30s under the structural equation modeling framework. As such, while we were not able to examine whether employment was in an integrative setting with the available data, we were able to capture the nature of competitive employment.

## Methods

### Data source

Our data source is the NLSY97, wherein a cohort of 9,984 youth aged 12 to 16 have been followed annually from 1997 to 2011, and biennially from 2013 on. Data are now available for the first 19 rounds of data collection (1997 to 2019). This sample includes a nationally representative cross-sectional sample of 6,478 youth and an oversample of 2,236 Black, Hispanic, and Latino youth. Even though parents provided information in Round 1 (Year 1997), youth participants are referred to as respondents to avoid confusion. The total retention rate went from 93% in Round 2 (Year 1998) to 75% in Round 18, fielded 2017–2018. Detailed information about sampling and data collection procedures can be found at https://www.nlsinfo.org/content/cohorts/nlsy97.

Sensory, mental, and physical conditions and their associated functional status were collected in 1997, 2002, 2007, 2008, 2009, and 2013. Parents reported in Round 1 (Year 1997) of data collection, and the youth respondents reported in the subsequent rounds. Sensory conditions included vision, hearing, and speech impairment; mental conditions included learning disability, emotional or mental problems, mental retardation, and eating disorder; physical conditions were indicated by bodily deformity, together with chronic health conditions such as asthma, diabetes, and cancer. For each condition, respondents were asked whether it limited one’s activities and when the condition was first noticed. In addition, participants were asked about general health limitations in 2007, 2008, 2009, 2010, 2011, 2013, 2015, and 2017. Participants reported whether they were limited in kind of work because of health or in amount of work because of health.

### Sample

The focus of the study was on the employment trajectory of youth with disabilities since high school. Therefore, we identified a sample that included respondents who reported a disability-related health condition at Round 6 (Year 2002) of the NLSY97 study when youth participants were at the age of 17 or above (all participants were at least 18 by 2003, but data on the disability-related health conditions were incomplete in 2003). In the NLSY97 survey, employment questions were asked since Round 1 for respondents 14 years and older. Given that respondents typically were not expected to complete high school until they turned at least 18 years old, we chose to examine employment starting from Round 7 (Year 2003) when all respondents were at least 18 years old. Following Mann and Honeycutt [20], we used the following questions at Round 6 (Year 2002) to identify the sample:

1. Have you ever had trouble seeing, hearing or speaking?
2. Have you ever had a part of your body that was deformed or missing?
3. Have you ever been diagnosed with any other chronic health condition or life-threatening disease such as [asthma, cardiovascular or heart condition, anemia, diabetes, cancer, epilepsy, HIV/AIDS, sexually transmitted disease other than HIV/AIDS, other]?
4. Have you ever had an eating disorder, a learning or emotional problem or a mental condition that has limited your ability to attend school regularly, do regular school work, or work at a job for pay?

Participants were included in the sample if they answered “Yes” to one or more of any of the four questions. This process resulted in an overall sample of 1,939 youths. Further, we excluded the 18 youths who reported a “mixed race” from subsequent analyses due to the extremely small cell size. The final analysis sample consisted of N = 1,921 youths.

### Measures

#### Competitive employment

To model longitudinal trajectories among youth with employment, we utilized a host of items to simultaneously assess two aspects of employment. In particular, we focused on wage-earning employment (employee-type jobs) and excluded other types of jobs such as freelance jobs, self-employment, and military service. The first aspect of employment we measured was the *stability of employment*. We computed the average hours worked per week from all employee-type jobs, as of either the job’s stop date or the interview date for ongoing jobs, to capture stability. Second, we measured the *quality of employment* using the hourly pay rate of the job, excluding overtime and performance pay, as of the date of interview or as of the job’s stop date. Using the consumer price index (CPI–CUUR0000SA0) data published by the U.S. Bureau of Labor Statistics, the hourly pay values were adjusted for inflation to year-2000 dollars.

#### Non-health related covariates

We examined the effect of several non-health-related factors on the youth’s employment trajectory. Specifically, the following factors were examined:

1. Demographic characteristics: sex (male vs. female) and race/ethnicity. Race/ethnicity was measured with three categories in the study: Black, Hispanic, and Non-Black/Non-Hispanic.
2. High school completion by age 20 years,
3. Certification from training experience, which is an indicator of whether the respondent has ever received a certificate or vocational license through any training program, and
4. Socioeconomic characteristics: residential mother’s education (below high school, high school, college) and household poverty ratio.

#### Condition and limitation status indicators

We examined the effect of health condition (i.e., disability type) and limitations separately.

1. Condition indicators: As in Mann and Honeycutt [20], the health condition reported in Round 6 (Year 2002) is categorized into three classes: “sensory” condition if answered “yes” to health condition question #1 above, “physical” condition if answered “yes” to health condition questions #2 or 3 above, and “mental” condition if answered “yes” to health condition question #4 above. Three corresponding binary (no/yes) condition indicators are created and used in the analyses.
2. Time-varying limitation status indicators: round-specific limitation status indicators that were available for Rounds 11 (Year 2007), 13 (Year 2009), 15 (Year 2011), 16 (Year 2013), 17 (Year 2015), and 18 (Year 2017). The youth was considered to have a limitation if they answered “yes” to at least one of two questions: a) Are you limited in the kind of work you do on a job for pay because of your health? and b) Are you limited in the amount of work you do because of your health?

### Analyses

#### Descriptive analyses

Descriptive analyses consisting of summary statistics and data visualizations were performed to describe patterns in the employment measures as well as the covariates. To explore the associations between the outcome variables and covariates, we reported and compared summary statistics for the outcome variables across the different levels of covariates (e.g., by type of health condition/limitation, sex, race/ethnicity, etc.).

#### Latent growth curve modeling

To identify and compare employment trajectories among youth with disabilities, we employed latent growth curve modeling (LGCM; also known as latent growth modeling–LGM), which is a flexible and powerful analytic tool that allows for investigating developmental patterns in longitudinal data, the between-individual and within-individual variability in the patterns, and how these patterns relate to external variables [e.g., 21, 22]. Since employment was represented by job quality (indicated by hourly pay) and job stability (indicated by weekly work hours), we first hypothesized and tested a multivariate LGCM [e.g., 23], wherein a latent construct “competitive integrated employment” was indicated by pay and stability, whose latent growth was in turn indicated by measurements of hourly pay and weekly work hours, respectively, over eight survey rounds. However, the multivariate LGCM failed to provide a good fit for the data as the two measures of employment, pay and stability, did not load on a common factor. Therefore, we opted for fitting a univariate LGCM for pay and stability separately.

**Fig 1:**
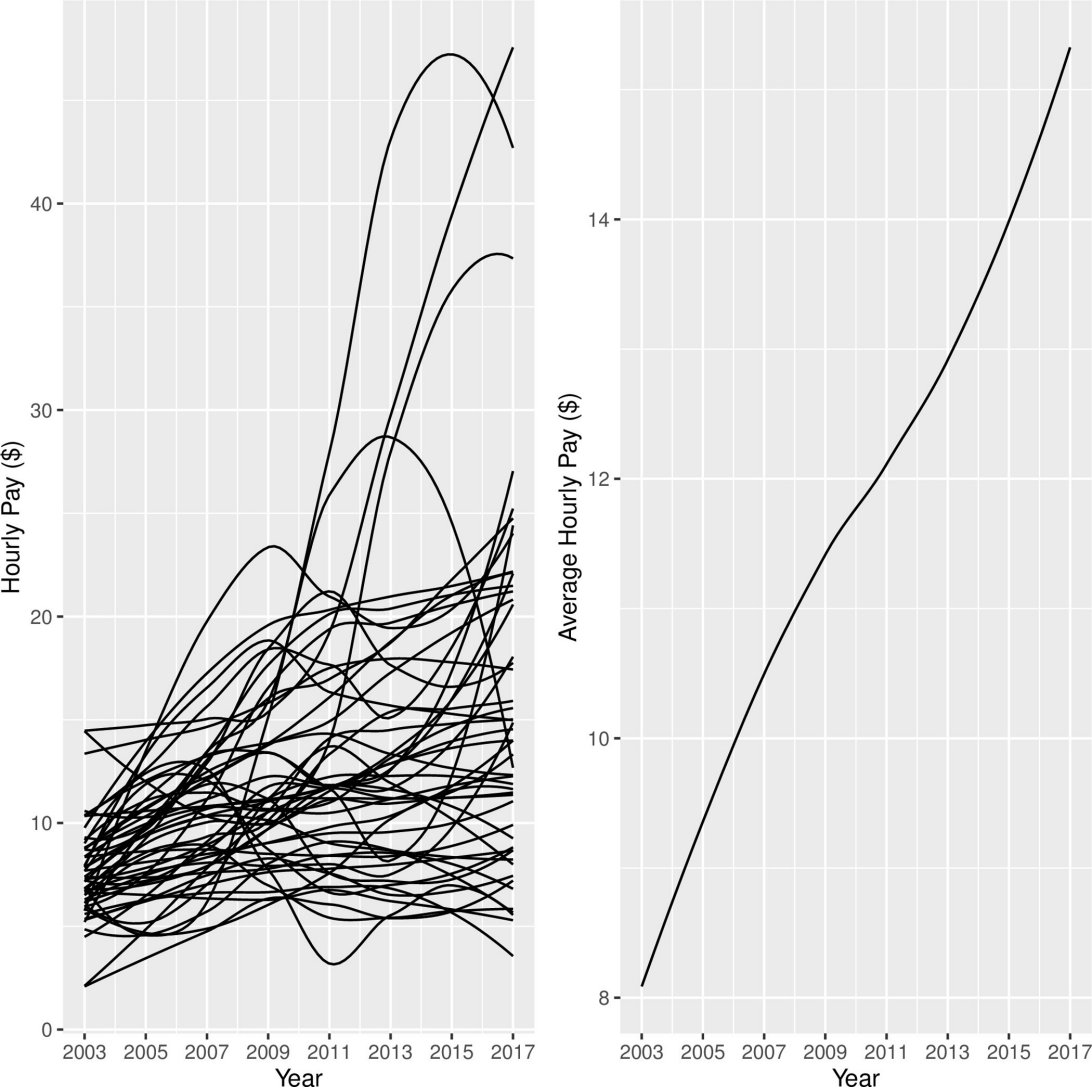
Hourly Pay from Employment (in 2000 US dollars) Trajectories: Random Sample of 50 Youth with Disabilities (Left) and Average Trend for Whole Sample (Right).

**Fig 2:**
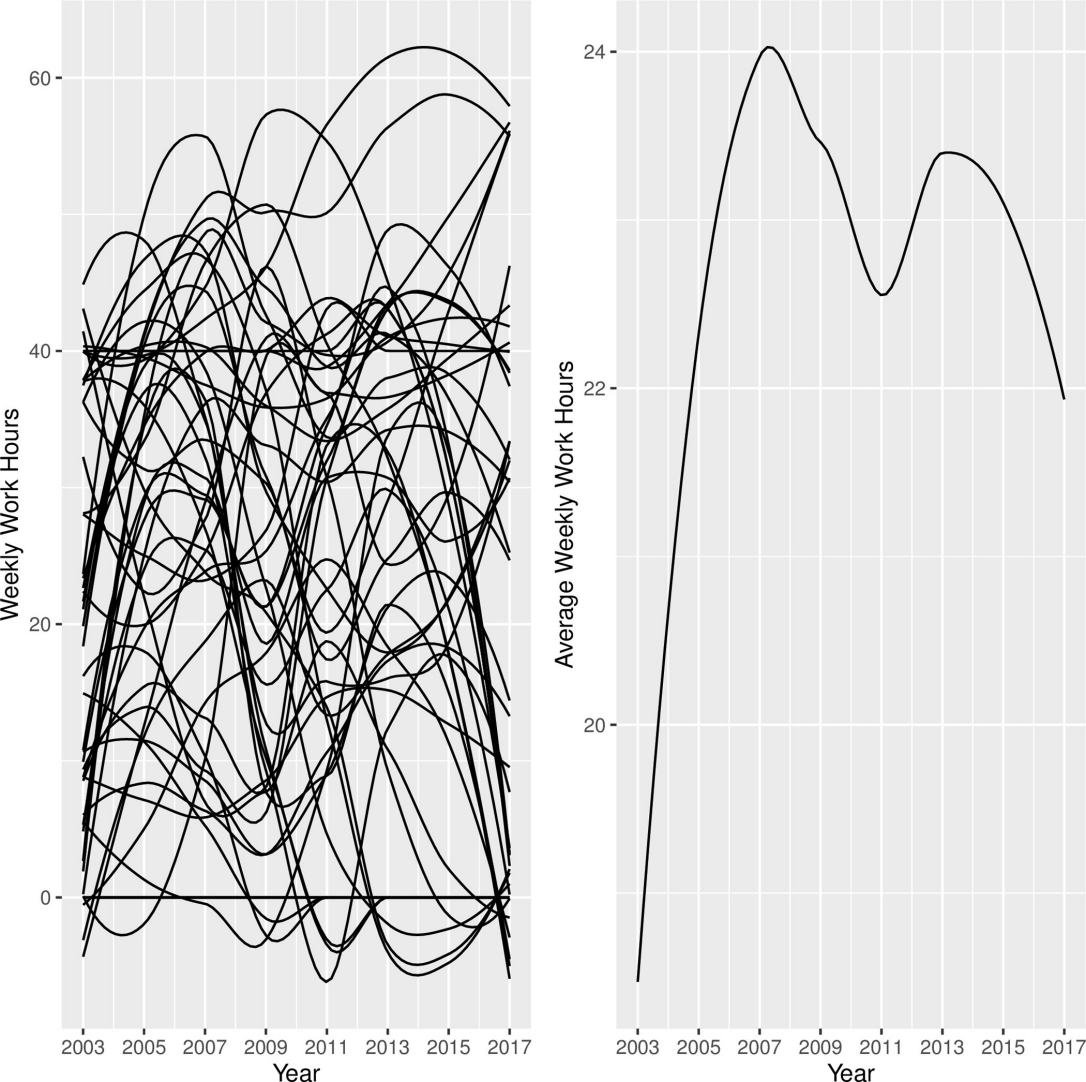
Weekly Work Hours Trajectories: Random Sample of 50 Youth with Disabilities (Left) and Average Trend for Whole Sample (Right).

Although the linear LGCM has been found to be successful in modeling growth in many applications, the functional form of growth is data-dependent and it should be determined based on theory, previous research, data visualizations and/or model selection procedures. Given the lack of literature on modeling employment trajectories among youth with disabilities, the appropriate functional form of growth was identified in this study by comparing goodness-of-fit indices for various candidate LGCM models. In order to gain an indication regarding the functional form of the growth model to be fitted, we visually inspected the trajectories of hourly pay and weekly work hours for a random subsample of 50 youth from the analysis sample (see Figs 1 and 2). As shown in the left panel in Figs 1 and 2, there was a great deal of variability among the 50 trajectories. Most importantly, these figures suggested that a linear change or even a curvilinear change might not be sufficient to capture the drop and the rise in the trajectories. Therefore, we fitted and compared three unconditional LGCMs; linear change, curvilinear change, and cubic change, for each of the two measures of employment. Once the functional form of growth was determined, we fitted conditional LGCMs to explore the effects of several covariates, including demographic, personal, familial and health characteristics, on the trajectories in employment measures for youth with disabilities.

**Fig 3:**
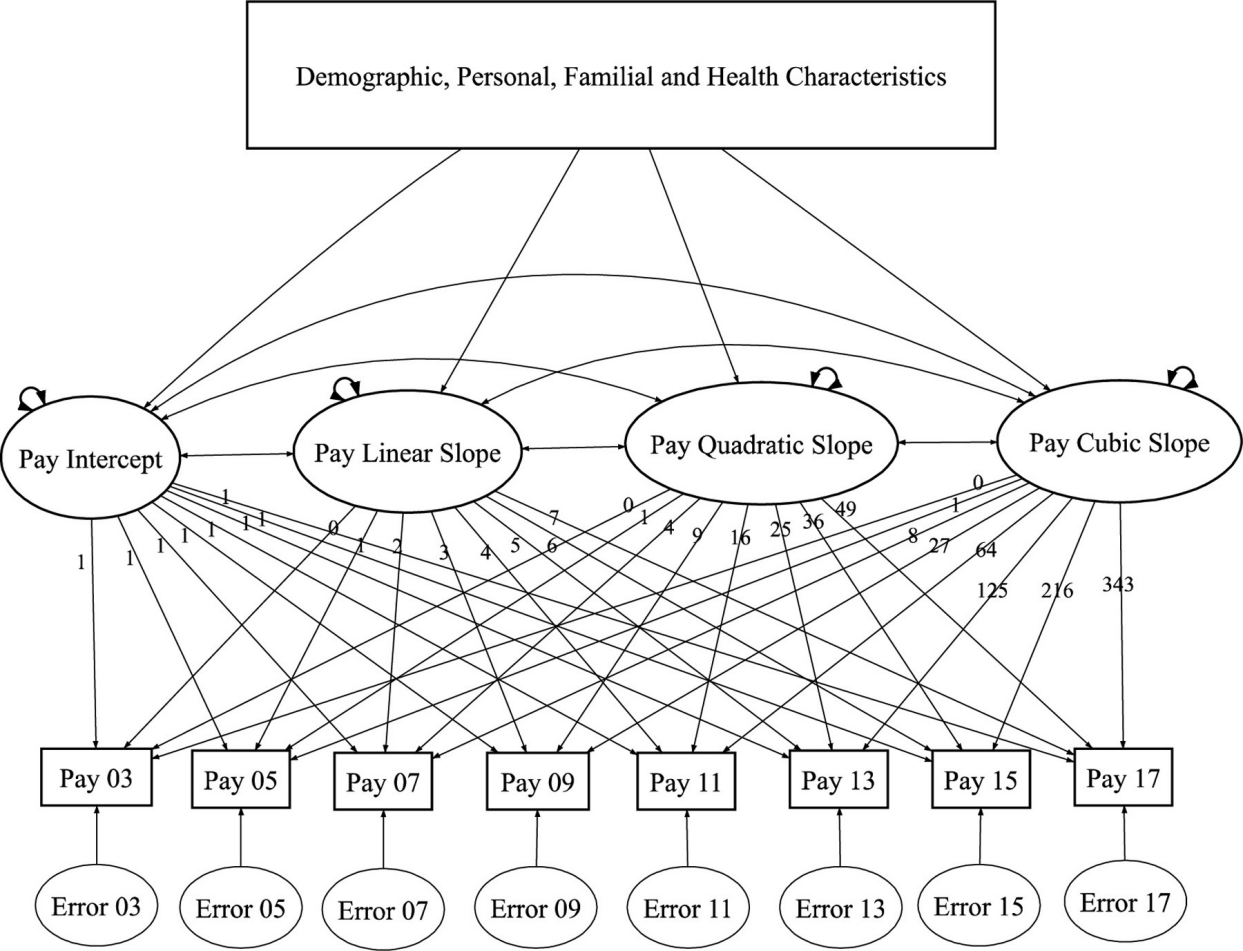
Graphical representation of the conditional univariate LGCM for pay from employment among youth with disabilities.

Fig 3 shows the cubic LGCM for hourly pay from employment (a similar representation applies for employment stability). The linear and quadratic LGCM have similar representations with modifications in the path specifications. Following the convention in structural modeling, in Fig 3 the observed variable (pay) is represented by “squares” and the latent (unobserved) variables are represented by “ellipses” [24]. Pay from employment at each round (Pay xx) was modeled as a function of the latent growth factors (intercept, linear slope, etc.) plus a random error (Error xx). The loadings for the intercept were fixed at 1 to assess the initial level (of job pay). The linear slope factor loadings were set as 0 for the baseline round (Round 7, Year 2003) to 7 for last round (Round 18, Year 2017) to represent a linear growth trend in hourly pay (note that the rounds are equally spaced with two years periods). The loadings of the quadratic slope factor were the squared loadings of the linear slope factor. Similarly, factor loadings of the cubic slope were the cubed loadings of the linear slope factor. The growth factors themselves were random variables with variances and covariances represented by the double-headed arrows. The means, variances and covariances of the growth factors are freely estimated.

Mathematically, the conditional cubic LGCM can be written as follows: for *i* = 1, 2, …,*N*,

$$y_{i}=\Lambda \beta_{i}+\varepsilon_{i},$$

$$\beta_{i}=\mu +\Gamma x_{i}+\xi_{i}.$$

Here, $y_{i}={\left(y_{i1},y_{i2},\ldots ,y_{iT}\right)}^{\prime }$ represents the vector of observed responses (e.g., hourly pay) for the *i*-th individual from *T* time points. $\beta_{i}={\left(\beta_{iI},\beta_{iL},\beta_{iQ},\beta_{iC}\right)}^{\prime }$ is a vector of latent growth factors (i.e., intercept, linear slope, quadratic slope, and cubic slope) with mean $\mu ={\left(\mu_{I},\mu_{L},\mu_{Q},\mu_{C}\right)}^{\prime }$. The factor loading matrix **Λ** is a *T* × 4 matrix whose *t*-th row is given by [1, (*t*—1),(*t*– 1)^2^,(*t*—1)^3^]defining the factor loadings for the growth factors at time *t* = 1, 2, …, *T*. Note that the time *t* is shifted by 1 to rescale the intercept factor to represent initial status (e.g., starting pay). **Γ** represents a matrix (4 × *q*) of regression coefficients of time-invariant covariate vector ***x***_*i*_ (*q* × 1). The errors (or unique factors) ***ε***_*i*_ are assumed to be normally distributed with mean **0** and common variance $\sigma_{e}^{2}.\xi_{i}={\left(\xi_{iI},\xi_{iL},\xi_{iQ},\xi_{iC}\right)}^{\prime }$ is a deviance vector, representing the deviation of the individual growth factor values from the factors means, that is distributed as multivariate normal with mean **0** and a 4 × 4 symmetric variance-covariance matrix ***Ψ*** whose diagonal elements are given by $\left(\sigma_{I}^{2},\sigma_{L}^{2},\sigma_{Q}^{2},\sigma_{C}^{2}\right)$ and off-diagonal elements are given by $\left(\sigma_{IL},\sigma_{IQ},\sigma_{IC},\sigma_{LQ},\sigma_{LC},\sigma_{QC}\right)$.

All analyses were conducted in the R software [25] version 4.1.3. Latent growth curve models were fit using the *growth* function in the *lavaan (Latent Variable Analysis)* package version *0*.*6–11* [26]. To account for non-normality of the response variables, especially the hourly pay (see Table 3), maximum likelihood estimation with robust (Huber-White) standard errors, scaled test statistics, and robust fit indices were obtained using the “estimator = MLR” option in the *growth* function [27]. The option “missing = ml.x” (alias “fiml.x”) was used to handle missing values in response and/or exogenous covariates when fitting the growth models. This is equivalent to the full information maximum likelihood approach where the likelihood is computed case by case using all data available for that case [26].

## Results

### Preliminary analyses

Table 1 provided summary statistics on two types of covariates included in the analyses of employment trajectories: non-health-related and health-related. The analysis sample of youths who reported some health condition in Round 6 (Year 2002) was slightly overrepresented by females (56% females vs 44% males). There was an over-representation of Black youth in the sample (26%), considering that Black or African American only accounted for 14.5% of the total 15- to 19-year-old population in the 2000 Census [28]. As expected, more participants reported physical or sensory disabilities than mental disabilities. Over the years, between 11% and 16% of participants reported that they were limited by their conditions to function in work or daily life.

**Table 1:** Descriptive statistics of the demographic, personal, familial, and health characteristics of the sample.

Covariate	N (%)	N Missing
*Non-health-related*		
Sex (2002)		0
Female	1078 (56.12)	
Male (Ref.)	843 (43.88)	
Race (2002)		0
Black	500 (26.03)	
Hispanic	356 (18.53)	
Non-black/Non-Hispanic (Ref.)	1065 (55.44)	
High School Diploma by Age 20 (2002)		460
No (Ref.)	1063 (72.76)	
Yes	398 (27.24)	
Education of Residential Mother (2002)		191
Below HS (Ref.)	374 (21.62)	
HS	574 (33.18)	
College	782 (45.20)	
Training Certification (2002)		618
No (Ref.)	368 (28.24)	
Yes	935 (71.76)	
*Health-related*		
Condition (2002) (Ref. = No)		
Sensory	823 (42.84)	0
Physical	1104 (57.47)	0
Mental	376 (19.59)	2
Has a Limitation? (Ref. = No) Yes		
2007	191 (10.95)	177
2009	203 (11.71)	187
2011	226 (13.16)	204
2013	255 (15.53)	279
2015	255 (15.53)	279
2017	230 (14.80)	367

We used Cramér’s V to assess the correlation among the covariates since all the covariates are categorical variables (see Table 2). Youth were more likely to have a high school diploma by age 20 when their residential mother had higher education levels. Non-black/Non-Hispanic youth were more likely than Black or Hispanic youth to obtain training certification or vocational licenses. Lastly, there was substantial overlap in disability types. That is, a number of youths had more than one type of disability.

**Table 2:** Cramér’s V correlation between covariates.

Covariate	Sex	Race	HS Diploma by Age 20	HGC RES MOM	HS TRN CERT	Sensory	Physical	Mental
Sex	–							
Race	0.017	–						
HS Diploma by Age 20	0.032	0.018	–					
HGC RES MOM	0.005	0.046	0.123^***^	–				
HS TRN CERT	0.016	0.242^***^	0.048	0.063	–			
Sensory	0.025	0.042	0.031	0.006	0.039	–		
Physical	0.041	0.025	0.038	0.010	0.046	0.562^***^	–	
Mental	0.011	0.081^*^	0.029	0.046	0.031	0.157^***^	0.261^***^	–

*Note*. ^*^Chi-square p-value < 0.01

^***^Chi-square p-value < 0.0001; HS = High School; HGC RES MOM = Highest Grade Completed by Residential Mother; HS TRN CERT = Earned Training Certification during High School.

As shown in Table 3, the average hourly pay (adjusted for inflation to year-2000 dollars) increased steadily over the years, but the distribution was skewed and non-normal, especially in the early rounds of 2003, 2005, and 2007, and there was a substantial amount of missing data in the wage variable across all waves. To weed out extremely high pay values, we used the 99th percentile of the hourly pay values ($56) as the cut-off for the inclusion of a reported pay value in the analysis. In contrast, the average work hours per week increased from 19 hours per week in 2003 to 25.8 hours per week in 2007, dropped a little afterwards, and remained between 22 and 24 hours in subsequent years (see Table 4). The number of work hours appeared to have symmetrical distribution across all waves and there were no missing data in this variable. Extreme numbers of weekly work hours, beyond the 99^th^ percentile (74 hours/week), were excluded from the analysis.

**Table 3:** Summary statistics of hourly pay (in year-2000 $) by survey year.

Year	Mean	Median	SD	Skewness	Kurtosis	Min	Max	N Missing
2003	8.1	7.2	4.22	3.72	27.86	0	52.0	372
2005	9.2	8.0	5.34	3.22	20.35	0	53.1	414
2007	10.3	9.0	5.78	2.28	11.33	0	52.1	371
2009	11.1	9.6	6.25	1.94	9.73	0	55.4	501
2011	11.6	9.9	6.93	1.94	8.83	0	53.7	545
2013	12.4	10.3	7.70	1.89	7.94	0	55.4	534
2015	13.2	10.9	8.11	1.76	7.16	0	54.6	567
2017	14.4	12.0	8.83	1.62	6.24	0	55.8	642

*Note*. 1,850 youths reported hourly pay in at least one of the 8 rounds. *Dollar figures are adjusted for inflation to year-2000 dollars*.

**Table 4:** Summary statistics of weekly work hours by survey year.

Year	Mean	Median	SD	Skewness	Kurtosis	Min	Max	N Missing
2003	19.0	16.0	18.60	0.33	1.70	0	74	0
2005	21.9	25.0	19.80	0.13	1.60	0	74	0
2007	25.8	35.0	20.70	-0.11	1.66	0	72	0
2009	22.7	28.0	20.50	0.09	1.51	0	72	0
2011	23.9	30.0	20.70	0.01	1.56	0	72	0
2013	23.6	30.0	21.00	0.05	1.53	0	72	0
2015	24.4	33.0	21.10	0.00	1.55	0	74	0
2017	22.2	25.0	21.70	0.19	1.48	0	72	0

*Note*. 1,921 youths reported weekly work hours in at least one of the 8 survey years.

Next, we examined the round-to-round correlations of hourly pay and weekly work hours (see Table 5). Not surprisingly, for both hourly pay and weekly work hours, each round had higher correlation with the round next to it than with rounds farther away.

**Table 5:** Round-to-Round pearson correlations of hourly pay (below diagonal) and weekly work hours (above diagonal).

Round	2003	2005	2007	2009	2011	2013	2015	2017
2003	**1.00**	*0*.*34*	*0*.*23*	*0*.*20*	*0*.*19*	*0*.*19*	*0*.*17*	*0*.*13*
2005	0.53	**1.00**	*0*.*37*	*0*.*32*	*0*.*28*	*0*.*30*	*0*.*28*	*0*.*21*
2007	0.31	0.48	**1.00**	*0*.*48*	*0*.*43*	*0*.*38*	*0*.*38*	*0*.*30*
2009	0.32	0.45	0.53	**1.00**	*0*.*54*	*0*.*44*	*0*.*41*	*0*.*33*
2011	0.26	0.34	0.45	0.64	**1.00**	*0*.*55*	*0*.*50*	*0*.*38*
2013	0.28	0.30	0.42	0.57	0.66	**1.00**	*0*.*59*	*0*.*48*
2015	0.21	0.30	0.40	0.52	0.63	0.73	**1.00**	*0*.*55*
2017	0.20	0.27	0.38	0.50	0.61	0.67	0.81	**1.00**

Before we formally constructed the measurement model, we examined the average hourly pay and weekly work hours from 2003 to 2017 by demographic subgroups (Tables 6 and 7). While the overall trends appeared to be similar between the subgroups (i.e., increasing hourly pay and stable work hours across waves), there seemed to be a difference in the initial levels of pay and work hours between female and male, among racial groups, and between those with a high school diploma versus those without, and such differences remained consistent across waves. Also, the rate of increase in hourly pay and work hours appeared to be different between subgroups. These preliminary analyses suggested that there was substantial variability in both the intercept and change components in characterizing the trajectories in employment outcomes.

**Table 6:** Trend in average hourly pay (in year-2000 $) from employment by covariates.

Covariate	Level	Year							
		2003	2005	2007	2009	2011	2013	2015	2017
Sex	Female	7.74	8.65	10.08	11.05	11.49	12.34	12.90	14.32
	Male	8.72	9.77	11.41	11.84	12.80	13.89	15.11	16.76
Race/Ethnicity	Black	8.22	8.51	9.73	10.45	11.11	11.76	11.79	12.41
	Hispanic	8.58	9.55	11.22	11.72	12.52	13.16	14.16	15.76
	Non-black/Non-Hispanic	8.02	9.26	10.84	11.65	12.28	13.44	14.55	16.38
HS Diploma	No	7.57	8.56	10.41	11.16	11.82	13.24	14.03	15.23
by Age 20	Yes	9.53	11.19	12.38	13.13	14.06	14.17	15.46	17.54
HGC RES MOM	Below HS	8.04	9.29	10.47	10.81	11.46	12.13	12.39	13.68
	HS	8.12	9.21	10.39	11.20	11.62	12.28	13.23	14.20
	COLLEGE	8.35	9.24	11.17	11.97	12.92	14.30	15.17	17.37
HS TRN CERT	No	8.24	8.99	10.28	10.39	11.20	12.79	13.75	15.26
	Yes	8.22	9.23	10.96	11.98	12.67	13.28	14.31	15.83
Sensory	No	8.06	9.28	10.82	11.62	12.18	13.22	14.34	16.10
	Yes	8.30	8.94	10.41	11.06	11.88	12.72	13.17	14.34
Physical	No	8.00	8.94	10.18	11.16	11.97	12.57	13.22	14.59
	Yes	8.27	9.27	10.98	11.55	12.11	13.32	14.29	15.91
Mental	No	8.24	9.28	10.88	11.54	12.26	13.31	14.08	15.63
	Yes	7.72	8.36	9.38	10.58	10.94	11.37	12.63	13.94

*Note*. HS = High School; HGC RES MOM = Highest Grade Completed by Residential Mother; HS TRN CERT = Earned Training Certification during High School. *Dollar figures are adjusted for inflation to year-2000 dollars*.

**Table 7:** Trend in average weekly work hours by covariates.

Covariate	Level	Year							
		2003	2005	2007	2009	2011	2013	2015	2017
Sex	Female	17.70	20.88	24.32	20.75	22.04	21.92	21.66	21.04
	Male	19.86	22.73	26.99	24.00	24.66	24.28	26.46	22.64
Race	Black	16.37	20.30	22.69	18.39	20.36	22.31	22.98	21.47
	Hispanic	18.85	21.73	25.09	21.82	24.08	22.67	21.87	19.73
	Non-black/Non-Hispanic	19.61	22.30	26.90	24.02	24.17	23.31	24.69	22.53
HS Diploma by Age 20	No	17.87	21.96	27.12	23.76	25.31	24.84	25.17	23.13
	Yes	25.03	25.8	28.39	25.29	25.90	26.45	26.57	24.08
HGC RES MOM	Below HS	17.76	20.55	22.46	18.75	20.19	19.91	20.88	19.64
	HS	19.82	23.03	27.08	22.54	22.24	22.75	23.23	21.55
	COLLEGE	18.97	21.67	26.65	24.12	25.74	25.11	26.02	23.65
HS TRN CERT	No	17.42	20.07	24.99	20.50	23.22	22.48	22.89	21.41
	Yes	20.44	24.07	28.29	25.46	25.27	25.45	27.29	24.25
Sensory	No	18.66	21.64	25.98	22.08	23.58	23.60	24.34	21.85
	Yes	18.57	21.71	24.76	22.23	22.59	22.01	22.88	21.56
Physical	No	17.76	21.05	24.05	21.66	22.68	21.13	22.89	20.20
	Yes	19.25	22.12	26.49	22.49	23.51	24.24	24.32	22.84
Mental	No	19.18	22.44	26.62	23.26	24.31	24.20	25.07	22.93
	Yes	16.31	18.50	20.75	17.57	18.45	17.71	18.17	16.81

*Note*. HS = High School; HGC RES MOM = Highest Grade Completed by Residential Mother; HS TRN CERT = Earned Training Certification during High School.

### Hourly pay trajectories

To model the trajectories of hourly pay for youth with disabilities, we first estimated three unconditional models with linear, quadratic, and cubic change, respectively. Latent variable means, variances, covariances, and fit indices for these models were presented in Table 8. All the latent variable means and variances in the model with cubic change were significantly different from 0. Moreover, it has the lowest normed chi-square (*χ*^2^/df = 29/22 = 1.32), the lowest RMSEA (0.020), and the highest CFI (0.997). Therefore, we decided that the cubic growth curve model provided the best fit to the data in describing change over time for hourly pay and proceeded with adding covariates to this model.

**Table 8:** Parameter estimates and fit indices for unconditional latent growth curve models fitted to hourly pay from employment for youth with disabilities.

	Growth Functional Form		
	Linear	Quadratic	Cubic
Latent variable means			
*μ*_*I*_	8.17 (0.102)^****^	8.14 (0.113)^****^	8.02 (0.101)^****^
*μ*_*L*_	0.83 (0.034)^****^	0.86 (0.076)^****^	1.48 (0.130)^****^
*μ*_*Q*_		-0.00 (0.010)	-0.27 (0.050)^****^
*μ*_*C*_			0.03 (0.005)^****^
Latent variable variances			
$\sigma_{I}^{2}$	10.71 (1.429)^****^	12.30 (1.894)^****^	12.27 (2.611)^***^
$\sigma_{L}^{2}$	1.33 (0.099)^****^	4.00 (0.466)^****^	9.89 (2.917)^***^
$\sigma_{Q}^{2}$		0.07 (0.009)^****^	1.40 (0.322)^****^
$\sigma_{C}^{2}$			0.01 (0.003)^****^
Latent variable covariances			
$\sigma_{IL}^{2}$	-0.58 (0.207)^**^	-2.07 (0.660)^**^	-2.10 (2.202)
$\sigma_{IQ}^{2}$		0.20 (0.078)^**^	0.24 (0.659)
$\sigma_{IC}^{2}$			-0.00 (0.056)
$\sigma_{LQ}^{2}$		-0.42 (0.060)^****^	-3.20 (0.939)^***^
$\sigma_{LC}^{2}$			0.29 (0.082)^***^
$\sigma_{QC}^{2}$			-0.14 (0.029)^****^
Fit indices			
*χ*^2^/df	151/31	89/27	29/22
CFI	0.945	0.972	0.997
TLI	0.950	0.971	0.996
AIC/BIC	69574/69646	69446/69540	69327/69449
RMSEA (95% CI)	0.067 (0.057, 0.078)	0.052 (0.040, 0.064)	0.020 (0.000, 0.037)
SRMR	0.046	0.031	0.020
GFI	0.975	0.984	0.993

*Note*. ^*^p < 0.05

^**^p < 0.01

^***^p < 0.001

^****^p < 0.0001. Standard errors are shown in parentheses. *χ*^2^ = rescaled (for non-normality) *χ*^2^ test statistic; df = degrees of freedom; CFI = robust comparative fit index and TLI = robust Tucker-Lewis index (see [29]); RMSEA = robust root mean square error of approximation (see [30]); AIC = Akaike information criterion; BIC = Bayesian information criterion; SRMR = standardized root mean square residual and GFI = Goodness of Fit Index (e.g., [31]). *Dollar figures are adjusted for inflation to year-2000 dollars*.

We adopted a hierarchical strategy to test which set of covariates best explained the trajectories of hourly pay for youth with disabilities. In Model 1, we only included disability type (health condition) as a covariate because disability status was the ultimate focus of this study and disability type has been shown to be a robust predictor of employment outcomes in the literature [2, 16]. In Model 2, we included disability type together with all basic demographic variables (sex, race, residential mother’s education level, and youth high school completion by age 20). Residential mother’s high school completion was considered a proxy for social class, and youth’s own high school completion was shown to be a critical factor for employment outcomes [16]. In Model 3, we included all covariates in Model 2 plus work training during high school and household poverty ratio. Although previous research demonstrated the importance of work experiences and training during high school for post-high school employment for youth with disabilities [4], the variable indicating training certification during or after high school had a substantial amount of missing data. As for the household poverty ratio, it was highly correlated with the residential mother’s education. Consequently, we added these two covariates in Model 3 to examine if they significantly improve model fit. In Model 4, we included all covariates in Model 3 plus limitations as a time-varying covariate. While we considered limitations critical for long-term employment [32, 33], we were cautious about including it as a covariate due to missing data and inconsistent data collection schedule for this variable.

According to the fit indices of the four conditional cubic growth curve models (Table 9), Model 2 demonstrated superior fit overall as compared to Model 3 and Model 4; in all fit indices except for AIC/BIC, Model 2 performed better than or equal to Models 3 and 4 while being more parsimonious than Models 3 and 4. Although Model 1 was comparable to Model 2 in most of the fit indices, Model 2 allowed for the examination of the effect of important demographic covariates (sex, race, residential mother’s education level, and youth high school completion by age 20) on the youths’ pay trajectories. As such, we retained Model 2 as the final model for describing the trajectories of hourly pay from employment for youth with disabilities.

**Table 9:** Fit indices for four conditional cubic growth curve models fitted to hourly pay from employment for youth with disabilities.

Fit indices	Model 1	Model 2	Model 3	Model 4
*χ*^2^/df	45/34	74/58	87/66	170/108
CFI	0.996	0.996	0.994	0.985
TLI	0.994	0.992	0.990	0.977
AIC/BIC	69034/69491	68173/68494	65486/65851	65288/65686
RMSEA (95% CI)	0.017 (0.000, 0.030)	0.014 (0.000, 0.024)	0.015 (0.002, 0.024)	0.020 (0.014, 0.025)
SRMR	0.017	0.013	0.013	0.031
GFI	0.992	0.990	0.990	0.986
# of Missing Patterns	190	363	602	737

*Note*. Model 1 included disability type (health condition) as covariate; Model 2 included disability type with additional demographic variables (sex, race, residential mother high school completion, and high school completion by age 20); Model 3 included all covariates in Model 2 plus work training during high school and household poverty ratio; Model 4 included all covariates in Model 3 plus limitations.

**Fig 4:**
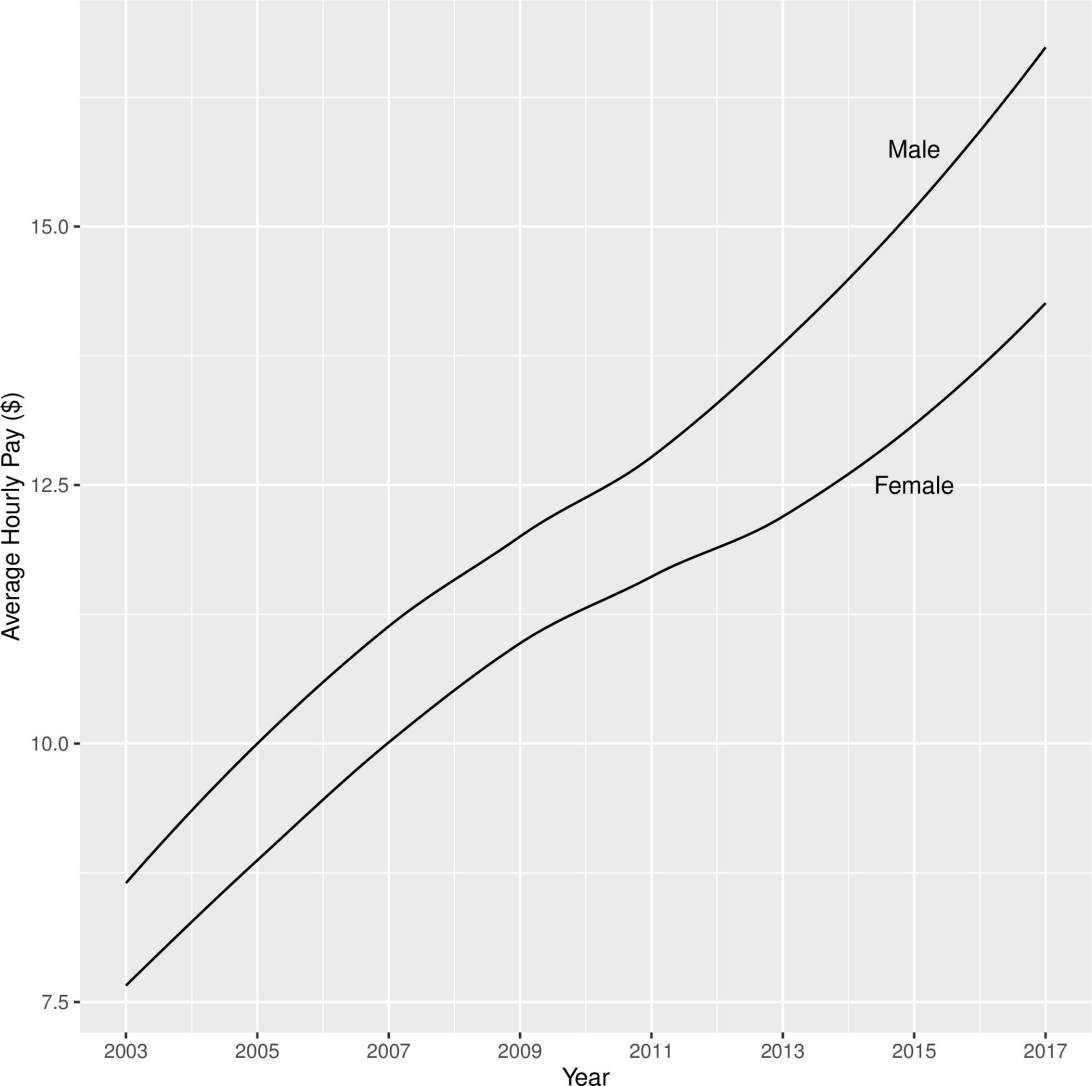
Trajectories of hourly pay (in year-2000 $) from 2003 to 2017 for male vs. female youth with disabilities.

**Fig 5:**
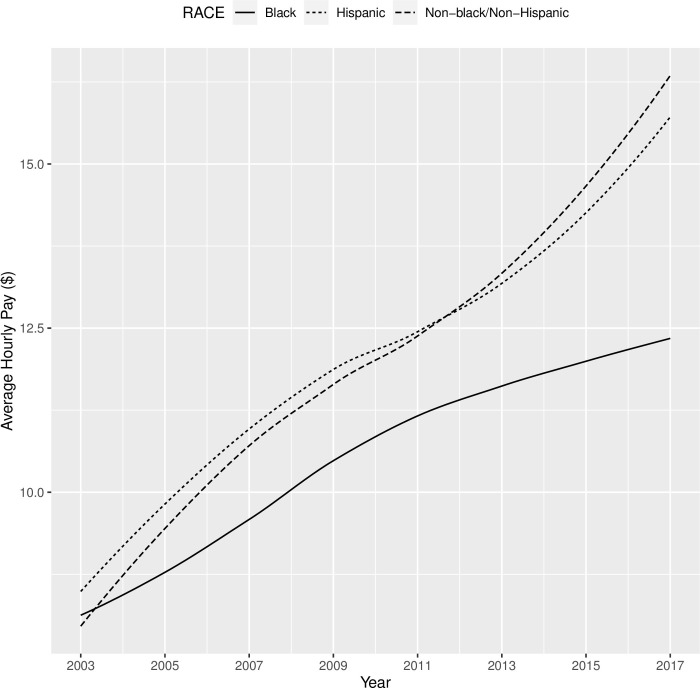
Trajectories of hourly pay (in year-2000 $) from 2003 to 2017 for Black vs. Hispanic vs. Non-Black/Non-Hispanic youth with disabilities.

**Fig 6:**
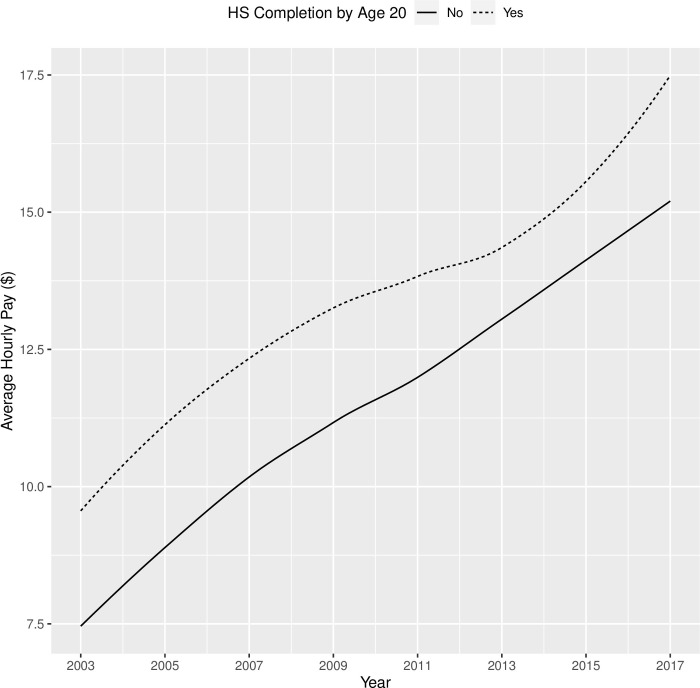
Trajectories of hourly pay (in year-2000 $) from 2003 to 2017 for youth with disabilities who completed high school by age 20 vs. those who did not.

**Fig 7:**
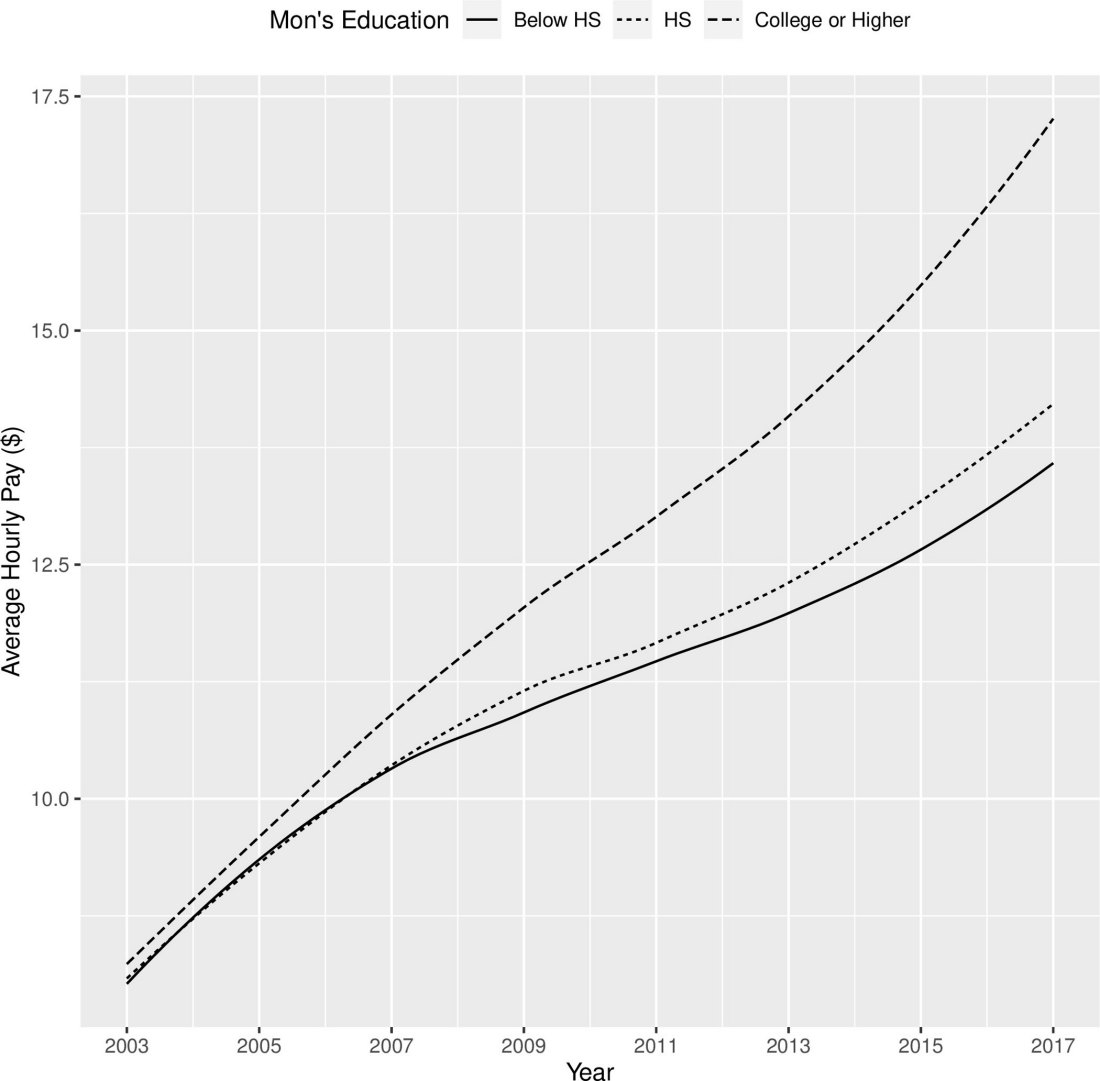
Trajectories of hourly pay (in year-2000 $) from 2003 to 2017 for youth with disabilities by the residential mother’s education level.

The results of Model 2 (parameter estimates, standard errors, and significance) were shown in Table 10. Participants made on average $7.83 per hour in 2003, and their hourly pay increased on average by $2.44 in each round. Additionally, both the quadratic and cubic component means were significantly different from 0. Youths with sensory disabilities had slower growth in pay than youth without sensory disabilities, and this contrast was consistent for mental and physical disabilities. As shown in Fig 4, male and female youths with disabilities had similar trajectories in terms of their hourly pay from 2003 to 2017, except that male youths started off with a higher pay ($1.15 per hour more) and retained this advantage over the years. Compared with non-Black/non-Hispanic youths with disabilities, Black youths with disabilities had a significantly lower hourly pay when they first transitioned out of high school in 2003 ($0.54 per hour less than their non-Hispanic/non-Black peers), and their hourly pay also appeared to have a slower growth (see Fig 5). When youths with disabilities completed high school by age 20, they had a much higher initial pay ($2.15 per hour more) when they transitioned into the workforce and retained this advantage until 2017 when they were around 30 years old, compared with their peers who did not finish high school by age 20 (see Fig 6). Lastly, residential mother’s education did not influence the hourly pay in 2003, but it appeared to make a difference in the rate of growth in subsequent years, according to Fig 7 which showed the bivariate association between residential mother’s education (below high school, high school, or college) and the hourly pay trajectory. Taking other covariates into account through the latent growth curve model, youths with disabilities whose residential mothers completed high school (but not college) had a significantly higher rate of linear growth in their pay from employment than their peers whose residential mothers did not complete high school. On the other hand, the model did not show a significant difference between the pay trajectories of youths with disabilities whose residential mothers did not complete high school and those whose residential mothers completed college (see Table 10).

**Table 10:** Parameter estimates for the selected conditional latent growth curve model (Model 2) for hourly pay from employment for youth with disabilities.

	Growth Factor			
	*μ*_*I*_	*μ*_*L*_	*μ*_*Q*_	*μ*_*C*_
Latent variable means	7.83 (0.425)^****^	2.44 (0.555)^****^	-0.51 (0.217)^*^	0.05 (0.021)^*^
Health condition				
Sensory	0.14 (0.332)	-1.17 (0.398)^**^	0.33 (0.147)^*^	-0.03 (0.014)^*^
Mental	-0.52 (0.291)	-1.23 (0.376)^**^	0.28 (0.148)	-0.02 (0.015)
Physical	0.40 (0.333)	-1.11 (0.400)^**^	0.30 (0.149)^*^	-0.03 (0.014)
Sex (Ref. = Male)				
Female	-1.15 (0.220)^****^	-0.12 (0.286)	0.06 (0.106)	-0.01 (0.010)
Race (Ref. = Non-Hispanic/Non-Black)				
Hispanic	0.18 (0.277)	0.21 (0.391)	-0.11 (0.152)	0.01 (0.015)
Black	-0.54 (0.254)^*^	-0.85 (0.333)^*^	0.26 (0.121)^*^	-0.03 (0.012)^*^
Residential Mother’s Education (Ref. = Below HS)				
HS	0.22 (0.287)	0.84 (0.371)^*^	-0.29 (0.145)^*^	0.03 (0.014)
College	0.22 (0.284)	0.54 (0.357)	-0.08 (0.141)	0.01 (0.014)
High School Diploma by Age 20 (Ref. = No)				
Yes	2.15 (0.320)^****^	0.46 (0.400)	-0.25 (0.146)	0.03 (0.014)

*Note*. ^*^p < 0.05

^**^p < 0.01

^***^p < 0.001

^****^p < 0.0001. Standard errors are shown in parentheses. *Dollar figures are adjusted for inflation to year-2000 dollars*.

### Weekly work hours trajectories

To model the trajectories of weekly work hours for youth with disabilities, we followed the same procedure as with hourly pay. We first estimated three unconditional models with linear, quadratic, and cubic change, respectively. Latent variable means, variances, covariances, and fit indices for these models were presented in Table 11. All the variances and covariances in the model with cubic change were significantly different from 0. Moreover, it has the lowest normed chi-square (*χ*^2^/df = 127/22 = 5.77), the lowest RMSEA (0.53), and the highest CFI (0.974). Therefore, we decided that the cubic growth curve model provided the best fit to the data in describing change over time for weekly work hours and proceeded with adding covariates to this model.

**Table 11:** Parameter estimates and fit indices for unconditional latent growth curve models fitted to weekly work hours of youth with disabilities.

	Growth Functional Form		
	Linear	Quadratic	Cubic
Latent variable means			
*μ*_*I*_	21.64 (0.369)^****^	19.67 (0.396)^****^	19.15 (0.420)^****^
*μ*_*L*_	0.39 (0.079)^****^	2.31 (0.242)^****^	3.72 (0.493)^****^
*μ*_*Q*_		-0.27 (0.032)^****^	-0.80 (0.166)^****^
*μ*_*C*_			0.05 (0.015)^**^
Latent variable variances			
$\sigma_{I}^{2}$	137.02 (7.175)^****^	135.52 (11.427)^****^	189.14 (19.331)^****^
$\sigma_{L}^{2}$	5.69 (0.394)^****^	43.82 (4.254)^****^	154.84 (21.210)^****^
$\sigma_{Q}^{2}$		0.69 (0.077)^****^	14.31 (2.131)^****^
$\sigma_{C}^{2}$			0.12 (0.019)^****^
Latent variable covariances			
$\sigma_{IL}^{2}$	-7.09 (1.442)^****^	-21.91 (6.038)^***^	-86.37 (18.573)^****^
$\sigma_{IQ}^{2}$		1.52 (0.720)^*^	21.04 (5.181)^****^
$\sigma_{IC}^{2}$			-1.65 (0.429)^***^
$\sigma_{LQ}^{2}$		-5.11 (0.549)^****^	-43.22 (6.427)^****^
$\sigma_{LC}^{2}$			3.42 (0.556)^****^
$\sigma_{QC}^{2}$			-1.25 (0.195)^****^
Fit indices			
*χ*^2^/df	475/31	189/27	127/22
CFI	0.887	0.959	0.974
TLI	0.898	0.958	0.966
AIC/BIC	131172/131244	130856/130950	130796/130918
RMSEA (95% CI)	0.092 (0.085, 0.099)	0.059 (0.051, 0.067)	0.053 (0.044, 0.062)
SRMR	0.085	0.034	0.029
GFI	0.960	0.985	0.990

*Note*. ^*^p < 0.05

^**^p < 0.01

^***^p < 0.001

^****^p < 0.0001. Standard errors are shown in parentheses. *χ*^2^ = rescaled (for non-normality) *χ*^2^ test statistic; df = degrees of freedom; CFI = robust comparative fit index and TLI = robust Tucker-Lewis index; RMSEA = robust root mean square error of approximation; AIC = Akaike information criterion; BIC = Bayesian information criterion; SRMR = standardized root mean square residual and GFI = Goodness of Fit Index.

We adopted the same hierarchical strategy used for modeling hourly pay to test which set of covariates best explained the trajectories of weekly work hours for youth with disabilities. The fit indices of four conditional latent growth curve models are given in Table 12. Similar to hourly pay, Model 2, which included disability type, sex, race, residential mother’s educational level, and youth high school completion by age 20 as covariates, had the best fit overall for the trajectories of weekly work hours; it provided excellent balance between quality of fit and parsimoniousness, while enabling us to examine the effects of important covariates (see Table 12). Therefore, we retained Model 2 as the final model for describing the trajectories of weekly work hours for youth with disabilities.

**Table 12:** Fit indices for four conditional cubic growth curve models fitted to weekly work hours of youth with disabilities.

Fit indices	Model 1	Model 2	Model 3	Model 4
*χ*^2^/df	149/34	183/58	194/66	421/108
CFI	0.972	0.972	0.971	0.938
TLI	0.958	0.951	0.949	0.906
AIC/BIC	130762/130951	129698/130020	126915/127282	126234/126634
RMSEA (95% CI)	0.044 (0.037, 0.051)	0.034 (0.029, 0.040)	0.033 (0.027, 0.038)	0.039 (0.035, 0.043)
SRMR	0.023	0.017	0.016	0.053
GFI	0.988	0.986	0.986	0.973
# of Missing Patterns	27	46	87	311

*Note*. Model 1 included disability type (health condition) as covariate; Model 2 included disability type with additional demographic variables (sex, race, residential mother high school completion, and high school completion by age 20); Model 3 included all covariates in Model 2 plus certification from training and household poverty ratio; Model 4 included all covariates in Model 3 plus limitations.

**Fig 8:**
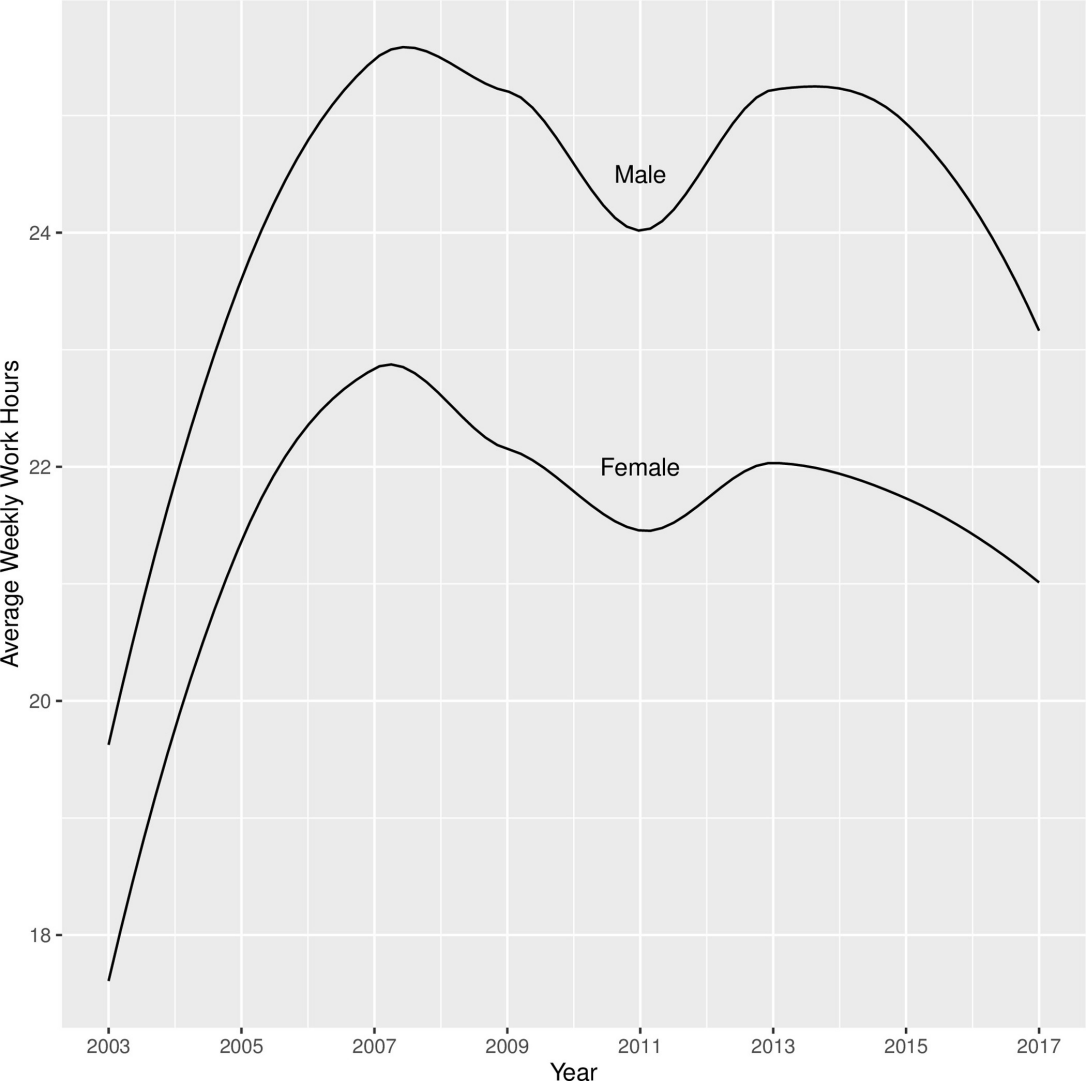
Trajectories of weekly work hours from 2003 to 2017 for male vs. female youth with disabilities.

**Fig 9:**
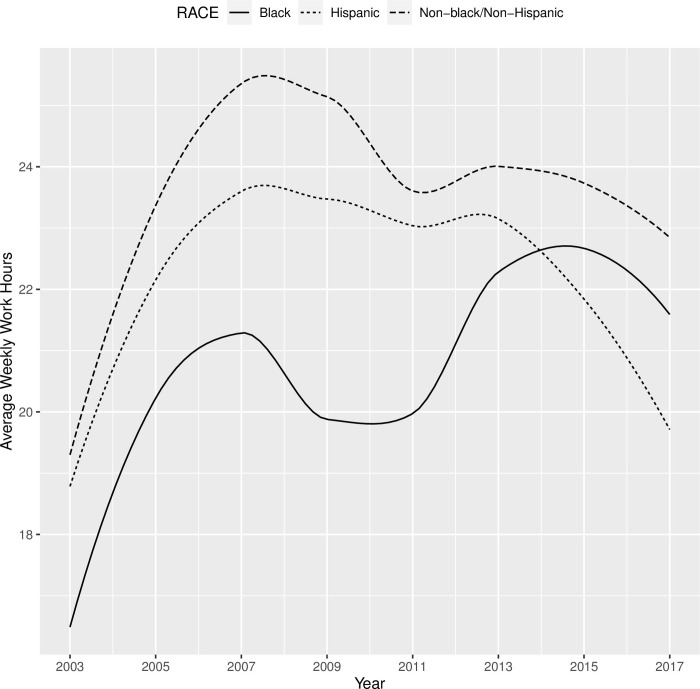
Trajectories of weekly work hours from 2003 to 2017 for Black vs. Hispanic vs. Non-Black/Non-Hispanic youth with disabilities.

**Fig 10:**
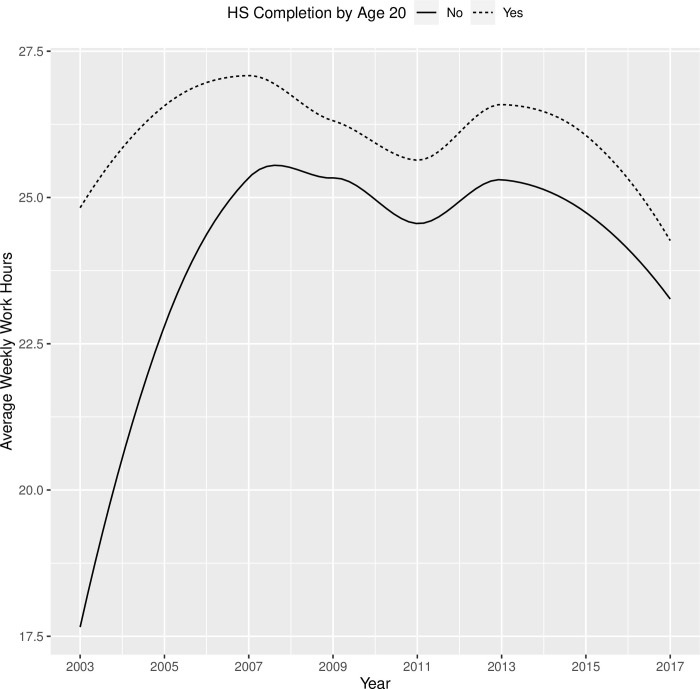
Trajectories of weekly work hours from 2003 to 2017 for youth with disabilities who completed high school by age 20 vs. those who did not.

As shown in Table 13, youths with disabilities worked for about 19 hours per week on average, and increased by about 5 hours in each round. Youths with mental disabilities started off working 3.26 fewer hours per week than youths without mental disabilities, but the growth patterns for the two groups were not significantly different. There was no significant difference between youths with and without sensory disabilities, or between youths with and without physical disabilities, in terms of baseline and growth in weekly work hours. As shown in Fig 8, male and female youths with disabilities had similar trajectories in terms of their weekly work hours from 2003 to 2017, except that male youths started off working 1.76 hours more per week and retained this advantage over the years. Compared with non-Black/non-Hispanic youths with disabilities, Black youths with disabilities had significantly lower hourly pay when they first transitioned out of high school in 2003 (2.8 fewer hours than their non-Hispanic/non-Black peers). Fig 9 showed that Black youths with disabilities appeared to experience a change in their trajectory and catch up with their peers in later rounds (2013+). However, none of the latent growth components (linear, quadratic, cubic) were significant once they were estimated in the presence of other covariates. Additionally, the work hours trajectories of Hispanic youths with disabilities were not significantly different from the trajectories of their non-Hispanic/non-Black peers. When youths with disabilities completed high school by age 20, they worked almost eight hours more per week than their peers who did not complete high school by this age. Although the latter group (those who did not complete high school by age 20) experienced a sharp increase in weekly work hours during the initial years of transition to the workforce, they were never able to catch up with their peers in terms of weekly work hours (see Fig 10). Furthermore, residential mother’s education did not significantly influence initial weekly work hours in 2003 or the growth in subsequent years. Finally, the downward trend that appears in Figs 8–10 around 2009 likely reflected the impact of the 2008 economic recession.

**Table 13:** Parameter estimates for the selected conditional latent growth curve model (Model 2) for weekly work hours of youth with disabilities.

	Growth Factor			
	*μ*_*I*_	*μ*_*L*_	*μ*_*Q*_	*μ*_*C*_
Latent variable means	18.88 (1.703)^****^	4.6 (1.929)^*^	-0.68 (0.649)	0.02 (0.06)
Health condition				
Sensory	-0.45 (1.097)	0.52 (1.251)	-0.57 (0.43)	0.07 (0.04)
Mental	-3.26 (1.156)^**^	-1.52 (1.276)	0.06 (0.44)	0.01 (0.041)
Physical	0.35 (1.133)	-0.28 (1.269)	-0.29 (0.434)	0.05 (0.041)
Sex (Ref. = Male)				
Female	-1.76 (0.835)^*^	0.22 (0.993)	-0.32 (0.336)	0.04 (0.031)
Race (Ref. = Non-Hispanic/Non-Black)				
Hispanic	-0.42 (1.158)	-0.43 (1.339)	0.34 (0.459)	-0.05 (0.043)
Black	-2.8 (0.997)^**^	-1.92 (1.194)	0.62 (0.4)	-0.04 (0.037)
Residential Mother’s Education (Ref. = Below HS)				
HS	1.65 (1.265)	1.43 (1.471)	-0.34 (0.5)	0.02 (0.046)
College	0.23 (1.199)	0.97 (1.391)	0.25 (0.474)	-0.05 (0.044)
High School Diploma AGE 20 (Ref. = No)				
Yes	7.71 (1.098)^****^	-4.16 (1.249)^***^	0.9 (0.419)^*^	-0.06 (0.039)

*Note*. ^*^p < 0.05

^**^p < 0.01

^***^p < 0.001

^****^p < 0.0001. Standard errors are shown in parentheses.

## Discussion

We assessed the feasibility of measuring competitive employment as a multidimensional, longitudinal outcome in a nationally representative sample of youth with disabilities using the NLSY97 data, in an attempt to align the measurement of this construct with its conceptualization. However, the multidimensional model where competitive employment was indicated by two lower level latent variables, pay and stability, was not supported by the data. Therefore, we assessed the longitudinal trajectories of pay and stability as separate constructs indicated by repeated measurements of hourly pay and weekly work hours, respectively. We found that there was substantial variability within the sample in both the intercept and slope of these two constructs, which were best captured by a cubic growth curve, and partially explained by health condition and several demographic variables, including sex, race/ethnicity, and high school completion by age 20.

Ideally, when youth with disabilities enter the workforce and obtain a position qualified as competitive integrated employment, they are able to work regular hours with steadily increasing pay over a long period of their adulthood. In reality, this is more of a conceptual framework than of a depiction of reality. Based on our findings from the NLSY97 data, these two elements of competitive integrated employment do not covary. That is, the trajectories of pay are not related to the trajectories of work hours. Research on the benefits of VR services commonly revealed that different types of VR services influence employment outcomes differently, depending on whether employment was indicated by having obtained employment at case closure [16, 34], wages or earnings [19, 32], or being employed after a certain period of time [4, 15]. Moreover, the magnitude of these benefits often varied as a function of disability type [3]. For example, a study on youth with autism in the VR system found that receiving job placement services was associated with higher likelihood of obtaining employment, yet postsecondary education most strongly predicted earnings [35]. Perhaps a multi-pronged approach, where multiple elements of employment are targeted, would be the most productive for long-term employment success beyond VR case closure for youth with disabilities.

In terms of hourly pay, we found that all participants experienced a steady increase over the course of 14 years since they entered the workforce. Nonetheless, participants who were female, Black, or did not complete high school by the age of 20 had a lower starting pay and a slower increase in pay longitudinally. These findings echo previous studies on a snapshot of employment earnings [e.g., 16]. In fact, higher education level (completing high school or college) seemed to consistently lead to higher wages [35, 36]. However, models that included training certification/licensure and time-varying limitations as covariates did not fit the data well, suggesting that the effects of these factors on hourly pay over the long term were negligible.

Although youth with disabilities, particularly women, were less likely than peers without disabilities to work 20 hours or more a week and more likely to be unemployed [37], there was a great deal of variability in terms of weekly work hours among youth in this sample. While some participants reported working 0 hours per week (i.e., unemployed), a lot of participants worked over 30 hours a week. Over the period between 2003 and 2017, weekly work hours demonstrated a sharp increase in the initial years, followed by a sharp decline, a small recovery, and then another decline. A similar set of predictors predicted weekly work hours as for hourly pay. Participants who were female, Black, or did not complete high school by the age of 20 started off with fewer work hours per week and retained this deficit throughout the years. These trends showed that job durability and stability were challenging for participants as they got older.

In this study, we investigated the long-term dynamics of employment outcomes among youth with disabilities by utilizing an innovative modeling technique to analyze longitudinal data with rich information collected from a nationally representative sample. However, several limitations must be noted, mostly because disability was not the primary focus of the NLSY97. First, there are a substantial amount of missing data in several key variables, including training certification and health limitations. Despite our efforts to handle missing data in our analyses, the amount of missingness certainly impacted model fit. Second, although the cubic model demonstrated a better fit than the linear and curvilinear models, the interpretation turned out to be challenging, particularly when the parameter estimates of the growth components beyond the slope were mostly insignificant in the conditional latent growth curve models. Finally, since this was intended to approach the concept of competitive integrated employment from a measurement perspective, we did not explore additional predictors or covariates.

Competitive integrated employment comprises of multiple components which should ideally be considered along a time dimension. Nonetheless, we found that two major components, hourly pay over time and weekly work hours over time, did not covary, even though their trajectories were associated with several demographic variables in a similar manner. Future studies need to assess validity of the measurement model with a different sample and incorporate another important component of competitive integrated employment, that is, whether work is carried out at an integrated setting.

## Supporting information

S1 Data (ZIP)
